# A simple modification of PCR thermal profile applied to evade persisting contamination

**DOI:** 10.1007/s13353-015-0336-z

**Published:** 2016-01-26

**Authors:** Michał Banasik, Anna Stanisławska-Sachadyn, Paweł Sachadyn

**Affiliations:** 1 Department of Molecular Biotechnology and Microbiology, Gdańsk University of Technology, Gdańsk, Poland; 2 Department of Biology and Genetics, Medical University of Gdańsk, Gdańsk, Poland

**Keywords:** False positive signal, No-template control, PCR contamination, qPCR, Quantitative PCR

## Abstract

The polymerase chain reaction (PCR), one of the most commonly applied methods of diagnostics and molecular biology has a frustrating downside known as the false positive signal or contamination. Several solutions to avoid and to eliminate PCR contaminations have been worked out to date but the implementation of these solutions to laboratory practice may be laborious and time consuming. A simple approach to circumvent the problem of persisting PCR contamination is reported. The principle of this approach lies in shortening the steps of denaturation, annealing, and elongation in the PCR thermal cycle. The modification leads to the radical decline of false positive signals obtained for the no-template controls without affecting the detection of target PCR products. In the model experiments presented here, the signal of negative control was shifted by about ten cycles up above those for the examined samples so that it could be neglected. We do not recommend this solution in PCR diagnostics, where the sensitivity of detection is of the highest priority. However, the approach could be useful to pass by the problem of persisting contamination in quantitative PCR, where the range of quantitation is usually much above the limits of detection.

**Electronic supplementary material:**

The online version of this article (doi:10.1007/s13353-015-0336-z) contains supplementary material, which is available to authorized users.

## Introduction

The polymerase chain reaction, PCR, the method which has revolutionized molecular biology and diagnostics offers the advantage of exponential signal amplification (Espy et al. 2006). The unsurpassed sensitivity of the method is connected with one of the most deteriorating limitations, which is the susceptibility to the so-called contaminations leading to false positive signals (Heid et al. 1996).

There are two main sources of PCR contaminations to be distinguished: (1) random contamination in the course of mixing reaction reagents and/or loading DNA templates and (2) the use of contaminated materials or reagents. Respecting good laboratory practices, such as, e.g., arranging separate post and pre-PCR rooms, can eliminate false positive signals resulting from PCR contaminations. The use of negative controls decreases the risk of releasing false positive results. Respecting good laboratory practice, though it is an obvious necessity in PCR diagnostics, may be difficult to follow in molecular biology laboratories, where PCR is one of many methods in use. PCR contamination often acts as a silent enemy, unreported in publications, but absorbing time and generating unproductive costs in research studies.

The idea of the solution proposed here emerged in the course of a research study where PCR quantitation was a crucial step. The progress of experiments was delayed because of persisting PCR contaminations. All good working laboratory practices were applied and all reagents and materials utilized for PCR were replaced, but false positive signals were still present in the no-template controls. The failure to eliminate the problem, prompted the need to search for a bypass. The principle of the solution was to modify the PCR thermal cycle in order to impair the sensitivity of detection leading to a decrease of the negative control signal but without affecting the detection of the specific product. This modification resulted in decreasing the signal for the no-template control by shifting C_t_ (cycle threshold) by about ten cycles up so that it could be neglected. This simple solution proved to be extremely helpful. We would like to report the concept, as we believe, it may save time and labour in qPCR examinations, whenever the quantitation, but not the detection limit is of priority.

## Materials and methods

### DNA templates and amplicons

The 69 bp templates and amplicons designated as R69 and O69 were obtained by hybridization of two pairs of complementary, HPLC purified synthetic, oligonucleotides (Table 1) (Genomed, Poland). The oligonucleotides (10 μM each) were hybridized in PCR reaction buffer (10 mM Tris–HCl (pH 8.8/25 °C), 50 mM KCl, 0.08 % (v/v) Nonidet P40) in a final volume of 100 μl by applying a thermal profile consisting of heating (92 °C/120 s), followed by two-stage cooling (65 °C/120 s and 25 °C/120 s). The amplicons derived from the human β-actin and the *GAPDH* gene transcripts were amplified from cDNA. The cDNA template was synthesized from total RNA extracted from EA.hy926 cells using 200 units of reverse transcriptase (SuperScript® III Reverse Transcriptase, cat. no. 18080044, ThermoFisher Scientific, USA), 100 pmoles of oligo dT_20,_ and 200 ng of template RNA in a final volume of 20 μl. The sequences of PCR primers and additional details on the templates and amplicons are given in Table 1.

**Table 1:** The list of PCR primers and amplicons

Amplicon	Primer forward/reverse (For/Rev) sequence	Template
R69, 69 bp DNA fragment	R69-For, CCCCCACCCACAGATCCA	Prepared from two complementary HPLC purified synthetic oligonucleotides listed in the bottom of the Table.
	R69-Rev,GGAGAAGAGGACAGCGGC	
O69, 69 bp DNA fragment	O69-For, CCACCACCCACTCACCAG	
	O69-Rev GAGGGCGCAGCAGAGAAG	
β144 - 144 bp fragment of human β-actin gene transcript (Genbank accession no. XM_006715764.1)	ACTB-For1, TGAGATTGGCATGGCTTTAT	Human cDNA synthesized from human total RNA extracted from EA.hy926 cells 2.8 x10^−3^ ng/μl (no-template control) and 2.8 ng/μl (positive control)
	ACTB-Rev1, GCCACATTGTGAACTTTGGG	
β 100–100 bp fragment of human β-actin gene transcript (Genbank accession no. XM_006715764.1)	ACTB-Forpub1, GATGAGATTGGCATGGCTT	Human cDNA synthesized from human total RNA extracted from EA.hy926 cells, 2.8 x10^−2^ ng/μl (no-template control) and 0.28 ng/μl (positive control)
	ACTB-Revpub1, CACCTTCACCGTTCCAGTTT	
GAPDH129 - 129 bp fragment of glyceraldehyde-3-phosphate dehydrogenase (*GAPDH*) gene transcript (Genbank accession no. NM_001289746.1)	GAPDH-For-3, CTCAGACACCATGGGGAAGG	Human cDNA synthesized from human total RNA extracted from EA.hy926 cells 5.6x10^−3^ ng/μl (no-template control) and 2.8 ng/μl (positive control)
	GAPDH-Rev-3, AGGTCAATGAAGGGGTCATT	
Oligonucleotides used to prepare O69 template	CCACCACCCACTCACCAGCTGTGCGACGAGCTGTGCCGCACGGTGATCGCACTTCTCTGCTGCGCCCTC GAGGGCGCAGCAGAGAAGTGCGATCACCGTGCGGCACAGCTCGTCGCACAGCTGGTGAGTGGGTGGTGG	
Oligonucleotides used to prepare R69 template	CCCCCACCCACAGATCCACTGTGCGACGAGCTGTGCCGCACGGTGATCGCAGCCGCTGTCCTCTTCTCC GGAGAAGAGGACAGCGGCTGCGATCACCGTGCGGCGCAGCTCGTCGCACAGTGGATCTGTGGGTGGGGG	

### qPCR reagents and conditions

**Fig. 1:**
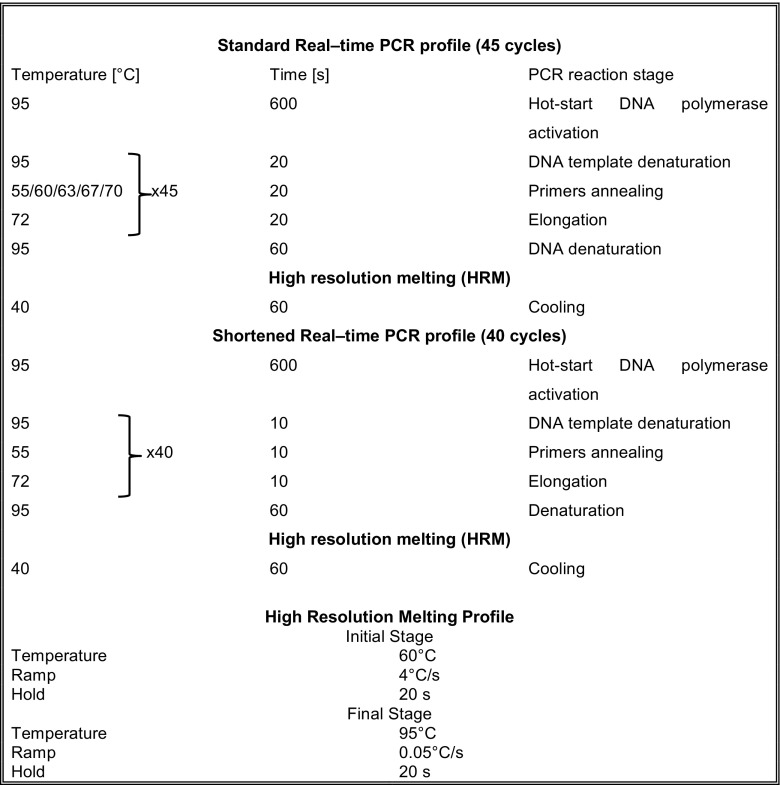
The “standard” and shortened real-time qPCR profiles. The “standard” and shortened real-time qPCR profiles. The principle of solution is compressing each step of PCR cycle from 20 to 10 s

qPCR experiments were performed in a final volume of 10 μl. Most qPCR reactions were performed with *Taq* DNA polymerase based SYBR-Green Master mix (2x HS PCR Master Mix SYBR®A, A&A Biotechnology, Poland, cat. No. 2017-100A). Alternatively, another master-mix was used for comparison (FastStart Essential DNA Green Master (Roche), cat. No. 06402712001). The forward and reverse primers were used at the final concentrations of either 0.5 μM or 0.1 μM each. The PCR thermal profiles are presented in Fig. 1. qPCR reactions were carried out using Nano LightCycler®Instrument (Roche Diagnostics). The C_t_ (cycle threshold) values and amplification efficiency values were calculated using LightCycler® Nano Software version 1.1 supplied by the manufacturer.

## Results

The method, proposed here, was used first in order to circumvent the problem of PCR contaminations occurring in the course of investigations on MutS protein (Sachadyn et al. 2000; Stanisławska-Sachadyn et al. 2005; Stanisławska-Sachadyn and Sachadyn 2005; Stanisławska-Sachadyn et al. 2006; Stanisławska-Sachadyn et al. 2003) where 69 bp amplicons were quantitated with real-time PCR in order to estimate the amounts of DNA bound by the protein. As mentioned above, all efforts, such as applying all good laboratory practices, replacement of PCR reagents and materials, and trying an alternative PCR master-mix, were ineffective to eradicate the persisting contaminations. The method introduced to pass the obstacle by is based on a modification of PCR thermal profile. The principle of the solution will be explained by presenting a series of qPCR experiments carried out for the 69 bp templates, designated further as R69 and O69 as the example. The PCR experiments were designed with regard to testing the impact of primer concentrations, annealing temperature, and the lengths of denaturation, annealing, and elongation steps in the thermal profile. All qPCR reactions reported below were performed in triplicates and each reaction was repeated three times. High resolution melting analyses were carried out to exclude the presence of non-specific PCR products.

**Fig. 2:**
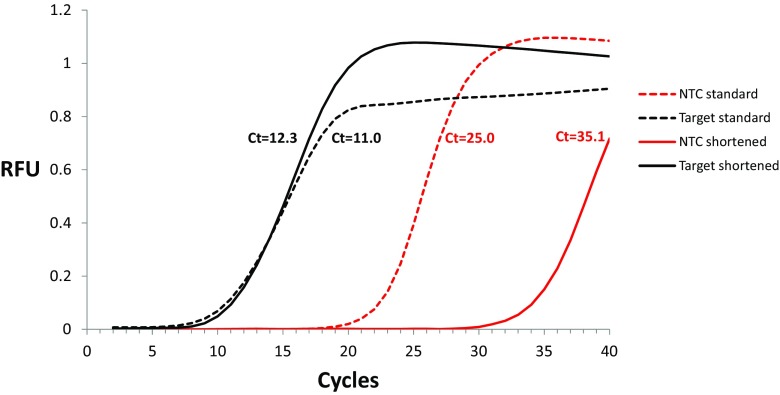
A representative example of C_t_ shift for no-template controls after applying “shortened” PCR profile. The PCR amplification was performed for the target DNA (R69) in the concentration of 10^−4^ μM using “standard” profile (black dashed curve) and shortened profile (black solid curve). The NTC (no-template control) PCR amplification curves are shown in red dashed curve (for the “standard” profile) and red, solid curve (for the shortened profile). The primer concentration in the amplification mixture was 0.1 μM, 40 amplification cycles were applied. *RFU* relative fluorescence units

**Fig. 3:**
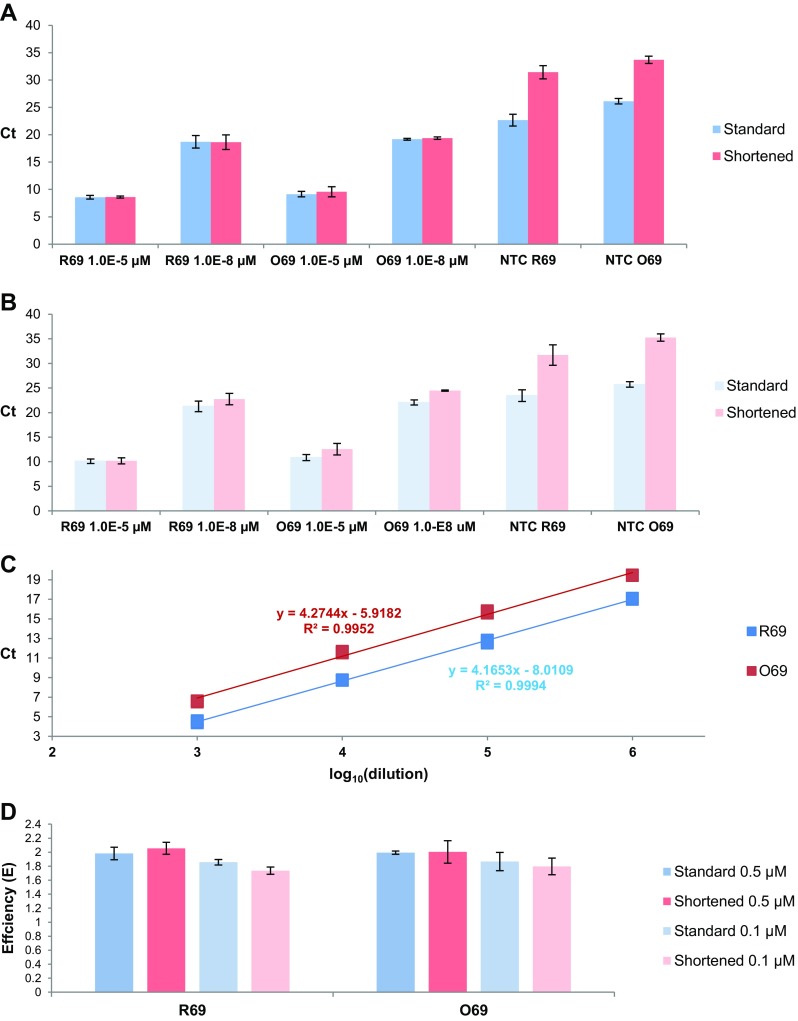
The impact of shortened PCR thermal profile on target detection and the levels of false positive signals. The C_t_ values obtained in the qPCR amplification for the 69 bp amplicons R69 and O69 (Table 1) and the no-template controls for the shortened and the “standard” thermal profiles (Fig. 1) using 0.5 μM **(a)** and **(b)** 0.1 μM primer concentrations. The standard curves of PCR reaction for the R69 and homoduplex O69 amplicons (0.1 μM primers concentration) in the shortened PCR profile **(c)**. The efficiencies (**e**) determined for qPCR amplifications of two 69 bp amplicons: R69 and O69 (Table 1) for the shortened and the “standard” thermal profiles (Fig. 1) using 0.5 and 0.1 μM primer concentrations **(d)**

With the aim of eliminating the false positive signals in the no-template controls a shortened thermal profile (Fig. 1) was applied. As compared to the “standard” thermal profile, the shortened one was trimmed by five cycles, but the principle of solution was compressing each step of PCR cycle - denaturation, annealing, and primer extension from 20 to 10 s. While the C_t_ values obtained for the positive controls in the qPCR amplification with the shortened thermal profile did not change significantly as compared to those obtained with the “standard” one, the C_t_ values for the no-template controls were significantly shifted in the instance of the shortened profile (Fig. 2), roughly by 7–9 cycles up (Fig. 3a and b). An analogical experiment to compare the “standard” and shortened thermal profiles was performed with fivefold lower primers concentrations, (0.1 μM). Similarly, as in the former experiment, the change of time/temperature profile led to similar results for the positive controls, whereas the C_t_ values obtained for the no-template controls were increased by about 8–10 cycles (Fig. 3b). The change of the thermal profile did not affect the linearity of PCR amplification (Fig. 3c). Also, the amplification efficiencies did not vary greatly with the change of the time/temperature profile (Fig. 3d). The decrease of primer concentrations caused a slight and statistically insignificant decline in the amplification efficiency for both thermal profiles and both amplicons. The detailed results are listed in Suppl. Table S2.

**Fig. 4:**
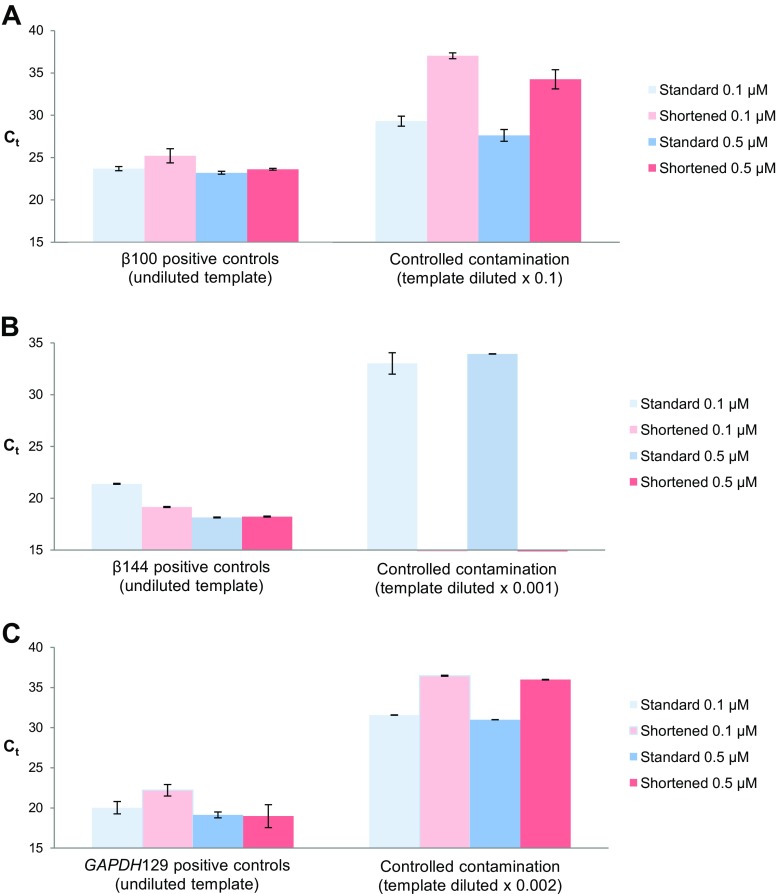
Shortening PCR thermal profile applied in the examination of gene expression. The C_t_ values obtained in the qPCR amplification using the shortened and the “standard” thermal profiles (Fig. 1) for the undiluted and the diluted cDNA templates corresponding to the positive and the negative controls (controlled contamination), respectively. The tested amplicons represented the β-actin and *GAPDH* gene transcripts (Table. 1). The final primers concentrations were either 0.1 or 0.5 μM each

Shortening PCR thermal profile solved the problem of contamination in the experiments presented above, so we decided to investigate if this approach is applicable to other models. As quantitating gene expression is one of the most important of PCR applications, the shortened thermal profile was applied in PCR amplifications of DNA fragments derived from the β-actin and *GAPDH* transcripts (Table 1). In order to model PCR contaminations, diluted cDNA templates were added to the negative controls (Table 1). Analogically, as in the experiments described above, the application of shortened PCR thermal profile led to a strong decrease (Fig. 4) or even disappearance (Fig. 4b) of contamination signals, while the signals representing the positive controls were either slightly shifted down or remained unchanged. The results were similar for the three tested amplicons, though the observed changes of C_t_ values varied dependent on the template dilution. It is worth noting that the application of the shortened profile had no effect on the detection sensitivity for the positive controls in the experiments with 0.5 μM primer concentrations, whereas the C_t_ values obtained for the artificially contaminated controls were strongly raised by approximately 5–6 cycles.

We would like to stress that we excluded the impact of annealing temperatures and the formation of non-specific PCR products as a possible source of no-template control signals. Several annealing temperatures were tested: 60, 63, 67, and 70 °C for the “standard” thermal profile (Fig. 1). Increasing annealing temperatures was ineffective to eliminate the false positive signals for the no-template controls. Raising annealing temperature led to moderate decreasing of the C_t_ values for both the positive and no-template controls of the R69 amplicon. In the case of the no-template controls for the O69 amplicons, the C_t_ values were shifted up by 3–4 cycles, while no impact on those for the O69 positive controls was observed (Suppl. Fig. S1). Changing annealing temperatures, even if it resulted in a moderate reduction of the false positive signal, was not sufficient to eliminate this deficiency.

The amplification of non-specific PCR products was excluded by high-resolution melting analysis (Suppl. Figs. S2 and S3), which confirmed the presence of the same amplicon in the positive and no-template controls. The C_t_ values for the no-template controls did not raise significantly with increasing annealing temperature (Suppl. Fig. S1), which supports the conclusion that primer-dimers formation did not contribute to increasing false positive signals in the presented experiments. The detailed results are listed in Suppl. Table S1.

## Discussion

Good laboratory practices are usually sufficient to prevent PCR contaminations in no-template controls. However, persisting PCR contaminations could be difficult to eradicate without paying the price of wasted reagents and delay in work progress.

As ultimate PCR sensitivity makes it susceptible to trace contaminations, we decided to search for a simple solution to reduce the excessive sensitivity of our assay. Increasing annealing temperature resulted in a moderate improvement but still no-template control signals interfered with the analysis. An essential advance was achieved by the application of PCR-thermal profiles with shortened steps of denaturation, annealing, and elongation. This approach resulted in a strong decline of the signal for the no-template controls without significant impact on the amplification from the target templates (within the examined range of concentrations). Then, the usefulness of the proposed modification of thermal profiles was evaluated for the amplicons used to quantitate gene expression, which is one of the most important of PCR applications.

Sub-optimal PCR conditions applied with regard to passing by the problem of intractable PCR contaminations may be obtained in other ways such as diluting DNA polymerase, exchanging reaction buffer, adding PCR inhibitors, and using lower concentrations of magnesium ions. However, such solutions would be much less convenient in qPCR than the simple modification of thermal profile, we tested. What is more, such methods would require time-consuming optimization and may result in a radical decline in the amplification of target templates. Also, simply decreasing the number of PCR cycles would be an inferior solution as it does not lead to separating the contamination signal from the target one.

To sum up, compressing the time of denaturation, annealing, and elongation in the PCR time-thermal profile is a simple and effective solution to evade the problem of false positive signals without essential decline in sensitivity. However, the final outcome of such modification strongly depends on the nature and concentration of the DNA template as well as the nucleotide sequence of the amplicon (cf. the differences between the C_t_ values obtained for β100 and GAPDH129 amplicons in different time/temperature profiles Fig. 4a and c).

## Conclusions

In principle, the problem of PCR contamination should be avoided by the implementation of good laboratory practices such as frequently changing gloves, using filter tips and decontamination reagents, arranging separate post and pre-PCR rooms, and when a contamination occurs, the change of PCR primers, reagents or amplicons, if possible (Dieffenbach and Dveksler 1993). If all such efforts are unsuccessful, we propose shortening the time of denaturation, annealing, and elongation in the PCR thermal profile. The proposed solution may be practical as long as the application of modified PCR thermal profile results in eliminating/decreasing the false positive signal of the no-template control without a significant impact on the sensitivity of detection. We do not recommend this solution for diagnostic laboratories, however, we think that it may be useful in research, in PCR quantitation experiments.

## Electronic supplementary material

Below is the link to the electronic supplementary material.

Fig. S1 The impact of annealing temperature on the PCR sensitivity and the levels of false positive signals.
The C_t_ values obtained in the qPCR amplification of 69 bp R69 and O69 amplicons (Table 1) and the no-template controls for different annealing temperatures for three DNA template concentrations using the “standard” thermal profile (Fig. 1). The final primers concentrations were 0.5 μM each (DOCX 63 kb)

Fig. S2 The representative melting profiles obtained for the R69 and O69 amplicons and the corresponding no-template controls (NTC) for 0.1 μM primer concentrations (DOCX 334 kb)

Fig. S3 The representative melting profiles obtained for the R69 and O69 amplicons and the corresponding no-template controls (NTC) for 0.5 μM primer concentrations (DOCX 334 kb)

Table S1 The C_t_ values obtained in the qPCR amplification for the R69 and O69 amplicons and no-template controls (NTC) using the "standard" and the shortened thermal profiles.
The experiments were performed in triplicates and each experiment was repeated three times (PDF 290 kb)

Table S2 The C_t_ values obtained in qPCR amplification for the R69 and O69 amplicons, and no template controls (NTC) using four annealing temperatures.
The experiments were performed in triplicates and each experiment was repeated three times (PDF 283 kb)

## Abbreviations

R69: DNA heteroduplex fragment, 69 bp in length containing TG mismatch in the position 35
O69: DNA homoduplex fragment, 69 bp in length
β144: 144 bp fragment of human β-actin gene transcript
β 100: 100 bp fragment of human β-actin gene transcript
GAPDH129: 129 bp fragment of glyceraldehyde-3-phosphate dehydrogenase (*GAPDH*) gene transcript.
qPCR: quantitative PCR
