# Supplementary material for: A simple modification of PCR thermal profile applied to evade persisting contamination
Source: J Appl Genet. 2016 Jan 26;57:409–15. doi: 10.1007/s13353-015-0336-z (PMC4963435; doi:10.1007/s13353-015-0336-z)
Supplement: Supplementary file 1 — The impact of annealing temperature on the PCR sensitivity and the levels of false positive signals. The Ct values obtained in the qPCR amplification of 69 bp R69 and O69 amplicons (Table 1) and the no-template controls for different annealing temperatures for three DNA template concentrations using the “standard” thermal profile (Fig. 1). The final primers concentrations were 0.5 μM each (DOCX 63 kb) [file 13353_2015_336_MOESM1_ESM.docx]

**A simple modification of PCR thermal profile applied to evade persisting contamination**

Journal of Applied Genetics

Michał Banasik^1^, Anna Stanisławska-Sachadyn^2^, Paweł Sachadyn^1^

^1^*Department of Molecular Biotechnology and Microbiology, Gdańsk University of Technology, Gdańsk, Poland*

*^2^Department of Biology and Genetics_,_ Medical University of Gdańsk, Gdańsk, Poland*

*to whom correspondence should be addressed: e-mail: psach@pg.gda.pl


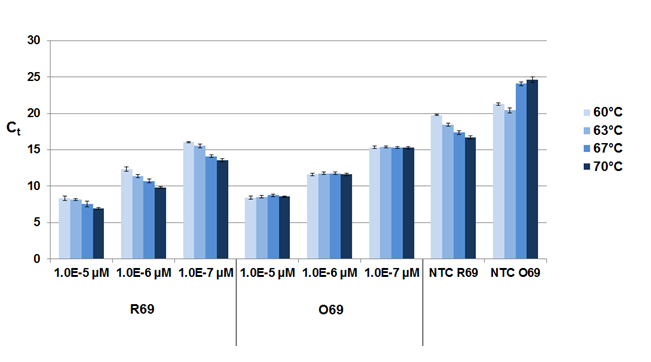


**Fig. S1. The impact of annealing temperature on the PCR sensitivity and the levels of false positive signals. The final concentration of primers in the reaction mixture was 0.5 µM.**
