# Supplementary material for: A simple modification of PCR thermal profile applied to evade persisting contamination
Source: J Appl Genet. 2016 Jan 26;57:409–15. doi: 10.1007/s13353-015-0336-z (PMC4963435; doi:10.1007/s13353-015-0336-z)
Supplement: Supplementary file 3 — The representative melting profiles obtained for the R69 and O69 amplicons and the corresponding no-template controls (NTC) for 0.5 μM primer concentrations (DOCX 334 kb) [file 13353_2015_336_MOESM3_ESM.docx]

**A simple modification of PCR thermal profile applied to evade persisting contamination**

Journal of Applied Genetics

Michał Banasik^1^, Anna Stanisławska-Sachadyn^2^, Paweł Sachadyn^1^

^1^*Department of Molecular Biotechnology and Microbiology, Gdańsk University of Technology, Gdańsk, Poland*

*^2^Department of Biology and Genetics_,_ Medical University of Gdańsk, Gdańsk, Poland*

*to whom correspondence should be addressed: e-mail: psach@pg.gda.pl


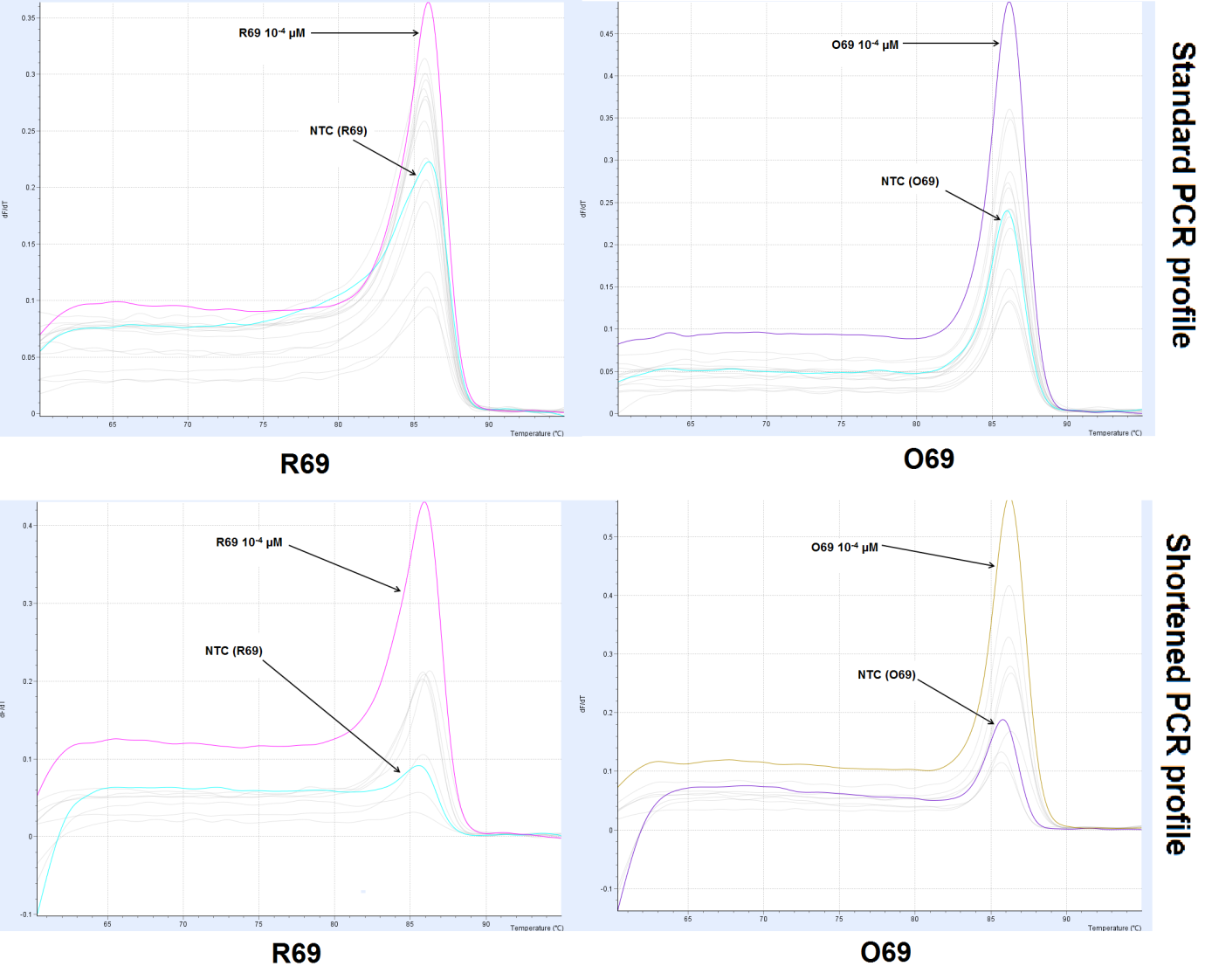


**Fig. S3.** **The representative melting profiles obtained for the R69 and O69 amplicons and the corresponding no-template controls (NTC).**

0.5 µM primer concentrations were used in PCR amplification.
