# Supplementary material for: A simple modification of PCR thermal profile applied to evade persisting contamination
Source: J Appl Genet. 2016 Jan 26;57:409–15. doi: 10.1007/s13353-015-0336-z (PMC4963435; doi:10.1007/s13353-015-0336-z)
Supplement: Supplementary file 4 — The Ct values obtained in the qPCR amplification for the R69 and O69 amplicons and no-template controls (NTC) using the "standard" and the shortened thermal profiles. The experiments were performed in triplicates and each experiment was repeated three times (PDF 290 kb) [file 13353_2015_336_MOESM4_ESM.pdf]

A simple modification of PCR thermal profile applied to evade persisting contamination

Journal of Applied Genetics

Michał Banasik[1], Anna Stanisławska-Sachadyn[2], Paweł Sachadyn[1]
[1]*Department of Molecular Biotechnology and Microbiology, Gdańsk University of Technology, Gdańsk, Poland*
[2]*Department of Biology and Genetics, Medical University of Gdańsk, Gdańsk, Poland*
*to whom correspondence should be addressed: e-mail: psach@pg.gda.pl

Table S1. The Ct values obtained in the Real–time PCR amplification for the R69 and O69 amplicons and no-template controls (NTC) using the "standard" and the shortened thermal profiles.
The experiments were performed in triplicates and each experiment was repeated three times.

Final primers' concentration 0.5 µM

	R69 10^{-5} µM	R69 10^{-8} µM	O69 10^{-5} µM	O69 10^{-8} µM	NTC R69	NTC O69		R69 10^{-5} µM	R69 10^{-8} µM	O69 10^{-5} µM	O69 10^{-8} µM	NTC R69	NTC O69
**Standard profile**	8,164	19,559	9,021	19,337	22,657	26,374	**Shortened profile**	8,922	18,616	11,072	19,577	31,042	34,612
	9,006	18,982	8,822	19,385	23,046	26,622		8,424	17,618	8,89	19,564	30,649	33,29
	9,109	19,658	8,939	19,377	22,924	26,486		8,762	22,005	8,829	19,625	30,293	33,949
	8,713	18,577	8,733	19,107	21,664	26,505		8,444	18,88	8,95	19,291	32,136	34,135
	8,471	17,762	8,806	19,145	22,537	26,492		8,697	18,142	8,899	19,495	32,591	33,994
	8,425	17,497	8,579	18,882	23,025	26,052		8,789	17,881	8,482	19,312	32,809	34,391
	8,763	(-)	9,835	19,052	20,502	25,596		8,598	18,66	10,405	19,277	29,189	32,777
	8,278	17,216	9,843	19,293	24,21	25,245		8,591	18,114	10,177	18,967	32,083	32,994
	8,327	20,45	9,642	19,063	23,426	25,679		8,359	17,88	10,429	19,212	32,022	33,084
arithmetic mean	8,584	18,712625	9,135555556	19,18233333	22,66567	26,11678	arithmetic mean	8,620666667	18,644	9,570333333	19,36888889	31,42378	33,69178
standard deviation	0,331007175	1,155164297	0,497075475	0,174511461	1,060955	0,497028	standard deviation	0,188643844	1,327803732	0,941370278	0,214111796	1,204153	0,665601

Final primers' concentration 0.1 µM

	R69 10-5 µM	R69 10-8 µM	O69 10-5 µM	O69 10-8 µM	NTC R69	NTC O69		R69 10-5 µM	R69 10-8 µM	O69 10-5 µM	O69 10-8 µM	NTC R69	NTC O69
**Standard profile**	10,728	22,386	10,206	22,289	22,631	26,492	**Shortened profile**	9,33	21,482	11,335	24,569	29,516	35,481
	10,592	21,939	10,204	22,641	23,23	26,345		9,414	21,361	11,511	24,571	29,681	36,563
	10,524	22,395	10,03	22,761	23,15	26,247		9,514	20,904	11,385	24,237	28,648	36,406
	9,952	19,668	11,001	22,345	22,723	25,811		10,344	23,661	12,303	24,491	30,98	35,088
	9,994	21,014	10,95	21,886	21,26	24,927		10,365	23,345	12,155	24,346	33,322	34,482
	10,193	22,18	10,71	22,299	24,451	25,675		10,412	23,393	12,224	24,47	32,654	34,783
	9,289	21,602	11,416	21,334	23,764	25,403		10,638	23,754	14,313	24,591	33,677	34,674
	9,767	19,8	11,723	21,514	25,037	25,06		10,665	22,831	13,756	24,639	32,419	34,829
	9,843	20,473	11,347	21,47	24,826	25,496		10,93	23,849	14,015	24,429	34,503	35,1
arithmetic mean	10,098	21,273	10,843	22,05988889	23,45244	25,71733	arithmetic mean	10,17911111	22,73111111	12,55522222	24,48255556	31,71111	35,26733
standard deviation	0,459977717	1,079637323	0,600614893	0,527176072	1,206559	0,557864	standard deviation	0,599503846	1,159818029	1,169661573	0,129215045	2,076701	0,74805
