# Supplementary material for: A simple modification of PCR thermal profile applied to evade persisting contamination
Source: J Appl Genet. 2016 Jan 26;57:409–15. doi: 10.1007/s13353-015-0336-z (PMC4963435; doi:10.1007/s13353-015-0336-z)
Supplement: Supplementary file 5 — The Ct values obtained in qPCR amplification for the R69 and O69 amplicons, and no template controls (NTC) using four annealing temperatures. The experiments were performed in triplicates and each experiment was repeated three times (PDF 283 kb) [file 13353_2015_336_MOESM5_ESM.pdf]

A simple modification of PCR thermal profile applied to evade persisting contamination

Journal of Applied Genetics

Michał Banasik[1], Anna Stanisławska-Sachadyn[2], Paweł Sachadyn[1]
[1]*Department of Molecular Biotechnology and Microbiology, Gdańsk University of Technology, Gdańsk, Poland*
[2]*Department of Biology and Genetics, Medical University of Gdańsk, Gdańsk, Poland*
*to whom correspondence should be addressed: e-mail: psach@pg.gda.pl

Table S2. The Ct values obtained in real-time PCR amplification for the R69 and O69 amplicons, and no template controls (NTC) using four annealing temperatures. The experiments were performed in triplicates and each experiment was repeated three times.

		Annealing temperature			
	DNA concentration [µM]	60°C	63°C	67°C	70°C
R69	1,00E-05	8,218	8,006	7,437	6,763
	1,00E-05	8,112	8,332	7,222	6,987
	1,00E-05	8,678	8,108	7,973	7,001
	Mean value	8,336	8,149	7,544	6,917
	Standard deviation	0,301	0,167	0,387	0,134
	1,00E-06	12,163	11,214	10,796	9,846
	1,00E-06	12,644	11,367	10,926	9,666
	1,00E-06	12,079	11,621	10,429	9,944
	Mean value	12,295	11,401	10,717	9,819
	Standard deviation	0,305	0,206	0,258	0,141
	1,00E-07	15,971	15,529	14,286	13,591
	1,00E-07	16,155	15,275	13,993	13,789
	1,00E-07	16,008	15,789	14,055	13,308
	Mean value	16,045	15,531	14,111	13,563
	Standard deviation	0,097	0,257	0,154	0,242
O69	1,00E-05	8,359	8,504	8,565	8,605
	1,00E-05	8,212	8,333	8,86	8,599
	1,00E-05	8,654	8,678	8,775	8,432
	Mean value	8,408	8,505	8,733	8,545
	Standard deviation	0,225	0,173	0,152	0,098
	1,00E-06	11,627	11,78	11,792	11,789
	1,00E-06	11,789	11,9	11,89	11,409
	1,00E-06	11,411	11,567	11,543	11,599
	Mean value	11,609	11,749	11,742	11,599
	Standard deviation	0,190	0,169	0,179	0,190
	1,00E-07	15,119	15,266	15,109	15,105
	1,00E-07	15,37	15,566	15,345	15,234
	1,00E-07	15,443	15,319	15,402	15,478
	Mean value	15,311	15,384	15,285	15,272
	Standard deviation	0,170	0,160	0,155	0,189
NTC R69		19,794	18,485	17,265	16,729
		19,809	18,2	17,678	16,937
		19,655	18,567	17,112	16,503
Mean value		19,753	18,417	17,352	16,723
Standard deviation		0,085	0,193	0,293	0,217
NTC O69		21,12	20,669	24,371	24,689
		21,22	20,555	23,876	24,914
		21,467	20,019	23,987	24,172
Mean value		19,753	18,417	17,352	16,723
Standard deviation		0,085	0,193	0,293	0,217
